# Haploidentical transplant with posttransplant cyclophosphamide vs matched related and unrelated donor transplant in acute myeloid leukemia and myelodysplastic neoplasm

**DOI:** 10.1038/s41409-023-02042-z

**Published:** 2023-07-21

**Authors:** Max J. Rieger, Sebastian M. Stolz, Antonia M. Müller, Rahel Schwotzer, Gayathri Nair, Dominik Schneidawind, Markus G. Manz, Urs Schanz

**Affiliations:** 1 Department of Medical Oncology and Hematology, University Hospital of Zurich, Zurich, Switzerland; 2 Department of Blood Group Serology and Transfusion Medicine, Medical University of Vienna, Vienna, Austria; 3 Department of Medicine II, University Hospital Tubingen, Tubingen, Germany

**Keywords:** Acute myeloid leukaemia, Stem-cell therapies

## Abstract

Hematopoietic cell transplantation from haploidentical donors (haploHCT) has facilitated treatment of AML and MDS by increasing donor availability and became more feasible since the introduction of post-transplant cyclophosphamide (ptCY). In our single-center retrospective analysis including 213 patients with AML or MDS, we compare the outcome of haploHCT (*n* = 40) with ptCY with HCT from HLA-identical MRD (*n* = 105) and MUD (*n* = 68). At 2 years after transplantation, overall survival (OS) after haploHCT was not significantly different (0.59; 95% confidence interval 0.44–0.79) compared to MRD (0.77; 0.67–0.88) and MUD transplantation (0.72; 0.64–0.82, *p* = 0.51). While progression-free survival (PFS) was also not significantly different (haploHCT: 0.60; 0.46–0.78, MRD: 0.55; 0.44–0.69, MUD: 0.64; 0.55–0.74, *p* = 0.64), non-relapse mortality (NRM) was significantly higher after haploHCT (0.18; 0.08–0.33) vs. MRD (0.029; 0.005–0.09) and MUD (0.06; 0.02–0.12, *p* < 0.05). Higher NRM was mainly caused by a higher rate of fatal infections, while deaths related to GvHD or other non-relapse reasons were rare in all groups. As most fatal infections occurred early and were bacterial related, one potential risk factor among many was identified in the significantly longer time to neutrophil engraftment after haploHCT with a median of 16 days (interquartile range; 14.8–20.0) vs. 12 days (10.0–13.0) for MRD and 11 days (10.0–13.0) for MUD (*p* = 0.01).

## Introduction

The introduction of allogeneic hematopoietic cell transplantation (HCT) from related, human leukocyte antigen (HLA) haploidentical donors (haploHCT) led to an increased availability of potential donors and thereby facilitated treatment of acute myeloid leukemia (AML) and myelodysplastic neoplasm (MDS) [1]. Previously avoided due to a higher risk of fatal infections and graft-versus-host disease (GvHD) [2–5], haploHCT has become a valid and widely used treatment option when combined with post-transplant cyclophosphamide (ptCY) [6, 7]. More recently, ptCy has also been shown to improve outcome for matched donor transplantation [8]. While initially mainly performed with bone marrow, subsequent studies demonstrated comparable results with mobilized peripheral blood stem cells (PBSC) [9]. Immunosuppression with ptCY together with mycophenolate mofetil (MMF) and a calcineurin inhibitor (CNI; ciclosporine A (CSA) or tacrolimus) minimizes the risk for acute and chronic GvHD and non-relapse mortality (NRM) early post-transplant by depletion of early alloreactive T cells [6, 10]. This is explained by their higher susceptibility to cyclophosphamide compared to resting T cells due to their cycling activity and proliferation, before they expand and infiltrate GvHD target organs [11]. Nevertheless, retrospective analyses in AML suggest that overall survival (OS) after haploHCT with ptCY still tends to be inferior compared with HCT from standard HLA- matched (min. 10/10) unrelated (MUD) or related (sibling) donors (MRD). However, published survival data are mostly from retrospective analyses and do not consistently reveal whether this trend is explained by a higher relapse rate or higher non-relapse mortality (NRM) [10, 12–14]. As a recent example, Mehta and colleagues highlighted that although haploHCT is associated with similar rate of relapse and severe GvHD, higher NRM was observed and primarily attributed to higher rates of fatal infections due to delayed T-cell reconstitution compared with MRD or MUD-HCTs [14–16]. To minimize infection risk, broad antimicrobial prophylaxis protocols have been implemented, generally combining antibiotics, antifungals, and antivirals, while substances vary substantially among different centers [15].

Here we report our single-center experience with haploHCT performed with ptCY, MMF and CNI as GvHD prophylaxis and compare it with MUD- and MRD-HCT performed with conventional immunosuppression. We demonstrate differences in NRM and cause of death distribution between groups, but not in OS and PFS and hypothesize on possible reasons for reported differences.

## Methods

### Patients

We retrospectively analyzed all patients over the age of 18 years diagnosed with AML or MDS who underwent first HCT between January 1st 2015 and December 31st 2020 at the Zurich University Hospital’s Department of Medical Oncology and Hematology in Zurich, Switzerland. We excluded transplantations from cord blood, unrelated HLA-mismatched donors, and manipulated grafts (such as ex-vivo CD34+ enrichment). We categorized patients according to donor type; related haploidentical, MRD and MUD matched at HLA-A*, B*, C*, DQB1* and DRB1* (minimum 10 of 10 HLA match), respectively. Original data was extracted continuously from electronic medical records by trained personnel and was later revised and analyzed by the authors.

### Endpoints

The primary objective of the study was to compare OS between groups. Secondary endpoints were PFS, NRM as well as causes of death and time from HCT to engraftment of neutrophils and platelets.

Furthermore, we documented prevalence of severe acute GvHD (aGvHD, grade II or higher) and peripheral whole blood chimerism on day 100 posttransplant. aGvHD was generally assessed and graded according to the consortium criteria published by Harris et al. [17].

Cause of death was categorized as either disease progression, GvHD, infection, or others (including graft failure (GF) and sinusoidal obstruction syndrome of the liver (SOS)). Since infections accompanied most deaths, death from infectious complications was only classified when there was no evidence of severe GvHD or morphological relapse. When both progression and severe GvHD and “other reasons” applied, progression was prioritized. Infectious deaths were further classified by the microorganism primarily responsible for the fatal outcome (bacterial, fungal, viral).

Time to engraftment of neutrophils (>1 × 10^9/L) and platelets (>50 × 10^9/L) was defined as time from HCT to the first day off at least three consecutive days above threshold without transfusions.

End of follow-up was June 30st 2021 representing at least 6 months follow-up after the last patient’s transplantation.

### Conditioning regimens

Patients who underwent haploHCT received either reduced intensity conditioning (RIC) with fludarabine (30 mg/m^2^ body surface, d-7 to −2) and busulfan (4 × 1 mg/kg body weight, d-3 & −2, concentration of steady state (CSS) adapted) or myeloablative conditioning (MAC) with busulfan (4 × 1 mg/kg d-7 to −4, CSS adapted) and cyclophosphamide (50 mg/kg, d-3 & d-2).

5 patients received an alternative RIC regimen with FluCy-sTBI (fludarabine 30 mg/m^2^, d-6 to −2; cyclophosphamide 14.5 mg/kg, d-6 and −5, and single-dose total-body-irradiation, 2 Gy on d-1). Immunosuppression after haploHCT included CSA (until d + 180), MMF (dose reduction from day +35) and ptCY (50 mg/kg, d + 3 & +4) according to published protocols [6].

Patient with MUD or MRD transplantation received either RIC with fludarabine, busulfan (dosage as above), and anti-thymocyte-globulin (ATG Neovii©, 10 mg/kg/d on days −4 to −1) or MAC with busulfan, cyclophosphamide, and ATG (s. above). G-CSF support was administered in all patients until neutrophil recovery >1 × 10^9/L. Immunosuppression (IS) in MRD and MUD-HCT was performed with CSA (dose reduction from day +100) and MMF (dose reduction from day+28 in MRD-HCT, d + 56 in MUD-HCT) after RIC, and CSA and methotrexate (MTX) after MAC (MTX 15 mg/kg bodyweight on d + 1, 10 mg/kg on d + 3, d + 6 and d + 11).

### Transplant and infection-related definitions

Antimicrobial prophylaxis was started after transplantation and always included valacyclovir and pneumocystis prophylaxis (usually trimethoprim-sulfamethoxazole) for at least 6 months. After haploHCT, antifungal prophylaxis with an azole (usually posaconazole) and antibiotics (usually levofloxacin) were added until IS cessation. Later, after its approval in Switzerland in 2020, cytomegaly-virus (CMV) prophylaxis with letermovir was introduced in high-risk HCT recipients.

In case of a suspected infection, empiric broad-spectrum antibiotic treatment with pseudomonas coverage was generally initiated (usually 4th generation cephalosporine or piperacillin/tazobactam). If imaging studies showed evidence of systemic fungal disease, antifungal prophylaxis was changed to another therapy, usually amphotericin B i.v. or another azole p.o. in the outpatient setting.

Valacyclovir at a prophylactic dose (500 mg bid) was dose escalated in case of signs of symptomatic herpes simplex or herpes zoster disease. CMV replication in the blood was monitored weekly by serum PCR, and treatment with valganciclovir was initiated in case of CMV disease or if copy numbers repeatedly reached >1000 IU/ml.

### Statistical analysis

Data were analyzed using *R Studio* [18] version 4.0.5. Comparisons between groups were analyzed using the Wilcoxon rank sum test (continuous data), Pearson’s chi-square test, or Fisher’s exact test (categorical data) as appropriate to test for statistical significance. Continuous data were summarized as median and interquartile range (IQR). Categorical data were summarized as numbers (n) and frequency (%). Figures were created using version 3.3.5 of *ggplot2* [19].

OS and PFS was estimated using the Kaplan-Meier method, log-rank test was used to evaluate differences between groups (packages *survival* and *survminer* [20], version 0.4.9). Surviving patients were censored at the date of last follow-up. PFS was defined as time from HCT to death from any cause or progression. Accordingly, patients who died before experiencing relapse were defined to have a competing event.

NRM was defined as death without relapse with relapse classified as a competing event. Probabilities of NRM were estimated with the use of cumulative incidence curves, Gray’s method was used to evaluate differences [21]. Missing data were dealt with by excluding patients from particular analyses if their file did not contain data for the required variables.

Hazard ratios for OS and PFS were evaluated using Cox proportional hazards regression analysis and Gray competing-risk regression analysis for NRM.

The cumulative incidence method was used to calculate incidence of causes of death over time from transplantation [21]. Here, no censoring for competing risks was performed. All *p*-values were adjusted (Bonferroni correction), *p* < 0.05 was considered statistically significant.

### Ethics approval

All patients provided informed consent according to the local institutions practice. The local ethics committee granted ethical approval for the study (BASEC No. 2022–00861) in accordance with the principles of the Declaration of Helsinki and its amendments [22].

## Results

### Patient characterisics

A total of 213 patients were included in this analysis, all underwent HCT as part of treatment for AML or MDS. 40 had a haploidentical donor, 105 a MUD and 68 a MRD.

Baseline characteristics are shown in Table 1. In all three cohorts, patients’ characteristics regarding age at diagnosis and transplantation, distribution of gender and ethnicity, Karnofsky performance status at transplantation, CD34 + PBSC count, and CMV risk status were similar. The haploHCT group had fewer patients with MDS and more with AML, fewer patients received myeloablative conditioning (MAC) and more were in morphologic complete remission (CR) at transplantation, although molecular remission rates and the distribution of ELN2022 risk groups at diagnosis were similar in all groups. As expected, donors in the MUD group were younger and more often male. The median duration of follow-up was 617 days (20.5 months, range 6.2–78.0 months).

**Table 1:** Baseline Characteristics.

Characteristics	Donor Group			*p*-value^b^
	Haploidentical, *N* = 40^a^	MRD, *N* = 68^a^	MUD, *N* = 105^a^	
Disease				0.041
AML	36 (90.0%)	47 (69.1%)	76 (72.4%)	
- ELN2022 adv. Risk	17 (47.2%)	20 (42.5%)	37 (48.6%)	
- ELN2022 int. Risk	15 (41.6%)	21 (44.6%)	28 (36.8%)	
MDS	4 (10.0%)	21 (30.9%)	29 (27.6%)	
R Age at Diagnosis (y)	56.3 (42.5, 64.3)	52.1 (41.5, 57.4)	53.0 (41.0, 60.5)	ns
R Age at TPL (y)	57.1 (43.3, 64.5)	53.1 (42.2, 58.8)	55.6 (41.4, 62.2)	ns
R Ethnicity				
White, non-hispanic	31 (77.5%)	54 (79.4%)	94 (89.5%)	
Latino or hispanic	7 (17.5%)	10 (14.7%)	8 (7.6%)	
Others	2 (5.0%)	4 (5.9%)	3 (2.6%)	
Conditioning Intensity (RIC)	32 (80.0%)	39 (57.4%)	71 (67.6%)	ns
Type of Conditioning				
MAC Regimens				
Bu-Cy-ATG	0 (0.0%)	21 (30.9%)	32 (30.5%)	
Bu-Cy	9 (22.5%)	2 (2.9%)	1 (1.0%)	
Flu-Cy-sTBI	5 (12.5%)	0 (0.0%)	0 (0.0%)	
other MAC	0 (0.0%)	5 (7.4%)	1 (1.0%)	
RIC Regimens				
Flu-Bu-ATG	0 (0.0%)	33 (48.5%)	68 (64.8%)	
Flu-Bu	26 (65.0%)	2 (2.9%)	2 (1.9%)	
other RIC	0 (0.0%)	5 (7.4%)	1 (1.0%)	
GvHD prophylaxis				
CNI/MMF	0 (0.0%)	40 (58.8%)	70 (66.7%)	
CNI/MTX	0 (0.0%)	24 (35.3%)	33 (31.4%)	
ptCY/CNI/MMF	40 (100.0%)	0 (0.0%)	1 (1.0%)	
other	0 (0.0%)	4 (5.9%)	1 (1.0%)	
Stem Cell Source				
BM (% patients)	2 (5.0%)	8 (11.8%)	3 (2.9%)	ns
No. CD34 + BMC (x10^6/ kg)	2.2 (1.7, 2.7)	1.0 (0.8, 1.7)	4.9 (2.9, 4.9)	
PBSC (% patients)	38 (95.0%)	60 (88.2%)	102 (97.1%)	ns
No. CD34 + PBSC (x10^6/ kg)	8.2 (7.1, 9.6)	7.3 (6.1, 9.4)	7.3 (5.6, 9.2)	
Relation D/R				
Sibling	0 (0.0%)	68 (100.0%)	15 (37.5%)	
Unrelated	0 (0.0%)	0 (0.0%)	105 (100.0%)	
Parent	5 (12.5%)	0 (0.0%)	0 (0.0%)	
Child	20 (50.0%)	0 (0.0%)	0 (0.0%)	
CMV-status D/R				
neg to neg	12 (33.3%)	21 (30.9%)	38 (36.5%)	
neg to pos	7 (19.4%)	11 (16.2%)	21 (20.2%)	
pos to neg	5 (13.9%)	4 (5.9%)	12 (11.5%)	
pos to pos	12 (33.3%)	32 (47.1%)	33 (31.7%)	
Gender D/R				
female to female	5 (12.5%)	18 (26.5%)	18 (17.5%)	
female to male	8 (20.0%)	13 (19.1%)	3 (2.9%)	
male to female	7 (17.5%)	9 (13.2%)	30 (29.1%)	
male to male	20 (50.0%)	28 (41.2%)	52 (50.5%)	
Donor Age	43.3 (31.8, 51.3)	50.1 (43.8, 60.3)	29.1 (23.8, 36.8)	<0.001
Morphological Remission at TPL				
CR	35 (87.5%)	44 (64.7%)	78 (74.3%)	
PR	3 (7.5%)	7 (10.3%)	9 (8.6%)	
R/R	1 (2.5%)	7 (10.3%)	4 (3.8%)	
untreated	1 (2.5%)	10 (14.7%)	14 (13.3%)	
Molecular Remission at TPL				
MinRD−	1 (2.5%)	3 (4.4%)	36 (34.3%)	
MinRD+	19 (47.5%)	38 (55.9%)	54 (51.4%)	
NA	20 (50.0%)	27 (39.7%)	15 (14.3%)	
Karnofsky at TPL > 0.8	35 (87.5%)	49 (72.1%)	89 (84.8%)	ns

*AML* acute myeloid leukemia, *ELN2022* European LeukemiaNet 2022, *adv.risk* adverse risk, *int.risk* intermediate risk, *MDS* myelodysplastic neoplasm, *R* recipient, *D* Donor, *TPL* transplantation, *RIC* reduced intensity conditioning, *Bu* Busulfan, *Cy* cyclophosphamide, *ATG* anti-thymocyte globulin, *Flu* fludarabine, *TBI* total body irradiation, *MAC* myeloablative conditioning, *GvHD* graft versus host disease, *CNI* calcineurin inhibitor, *MMF* mycophenolate mofetil, *MTX* methotrexate, *ptCY* posttransplant cyclophosphamide, *BMC* bone marrow cells, *PBSC* peripheral blood stem cells, *CMV* cytomegalovirus, *TPL* transplantation, *CR* complete remission, *PR* partial remission, *R/R* relapsed/refractory, *MinRD* minimal residual disease.

^a^*n* (%); Median (IQR).

^b^Pearson’s Chi-squared test; Kruskal-Wallis rank sum test; Fisher’s exact test.

### Survival, PFS and NRM

**Fig. 1:**
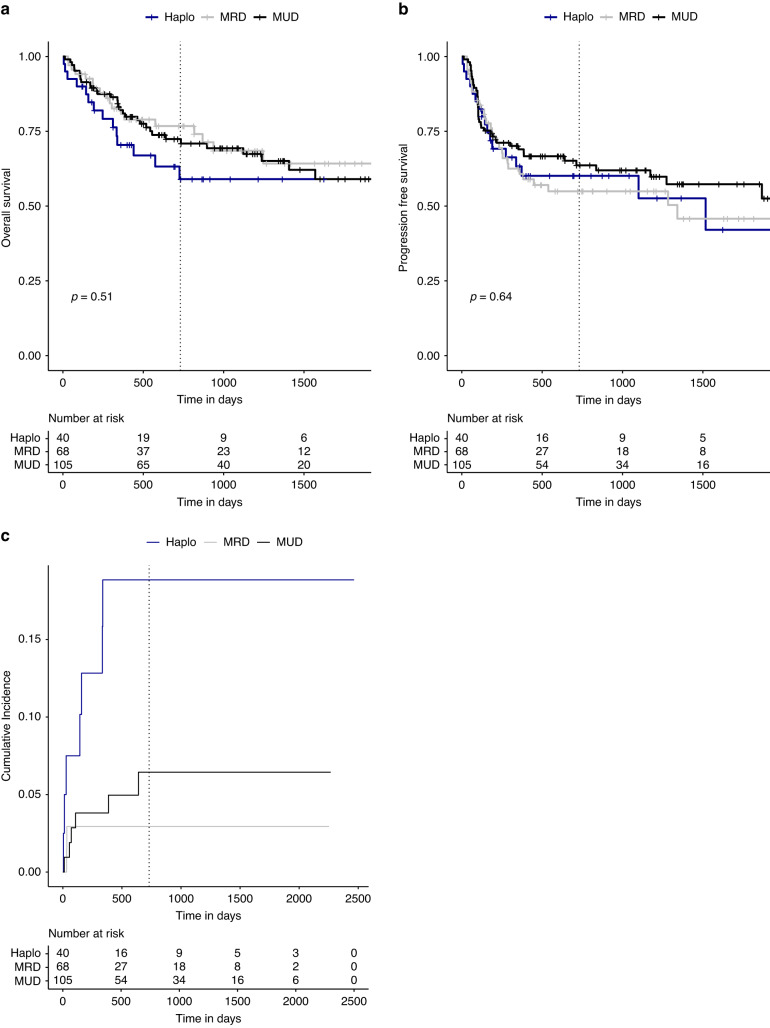
Transplant outcomes. **a** Overall survival, **b** progression-free survival, and **c** non-relapse-mortality. Vertical dotted lines indicate 2 years (day 730) from transplantation.

Differences in OS grew over time from transplantation but did not reach statistical significance. At 2 years after HCT, probability of OS after haploHCT was 0.5 (95% confidence interval; 0.44–0.79) compared to 0.77 (0.67–0.88) after MRD and 0.72 (0.64–0.82) after MUD transplantation (Fig. 1a, Log-rank; *p* = 0.51). In the univariate analysis, hazard ratio for OS at 2 years was 0.54 for MRD (0.26–1.14) and 0.62 for MRD (0.32–1.19) compared to haploHCT.

PFS also was not significantly different between groups. At 24 months, probability of PFS after haploHCT was 0.60 (95% CI; 0.46–0.78), 0.55 (0.44, 0.69) after MRD-HCT, and 0.64 (0.55–0.74) after MUD-HCT (Fig. 1b, Log-rank; *p* = 0.64). HR for PFS at 2 years was 1.08 (0.58–2.03) for MRD and 0.87 (0.48–1.59) for MUD compared to haploHCT.

However, NRM was significantly higher after haploHCT with a cumulative incidence at 2 years of 0.18 (95% CI, 0.08–0.33) versus 0.029 (0.005–0.09) after MRD-HCT and 0.06 (0.02–0.12) after MUD-HCT (Fig. 1c, *p* = 0.01). HR for NRM at 2 years was 0.31 (0.10–0.92) for MRD and 0.31 (0.03–0.80) for MUD compared to haploHCT.

### Causes of death

**Fig. 2:**
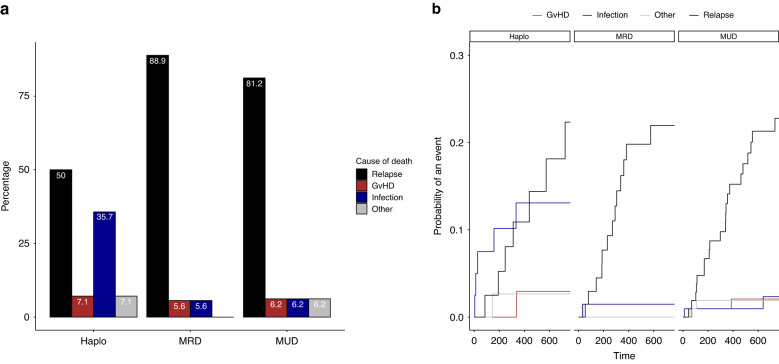
Causes of death within groups. **a** Distribution (%) at 2 years. **b** Cumulative incidence over time within the first 2 years from transplantation. “Other” causes of death include graft failure and sinusoidal obstruction syndrome.

Cumulative incidence of causes of death demonstrate higher NRM after haploHCT is mainly caused by a higher rate of fatal infections (Fig. 2b), with most infectious deaths occurring within the first 100 days after transplantation. At 2 years, cumulative incidence of death due to infection reaches 0.13 (95% CI; 0.046–0.259) after haploHCT versus 0.014 (0.0012–0.070) after MRD and 0.023 (0.0042–0.075) after MUD transplantation. Meanwhile, cumulative incidence of deaths due to relapse reaches 0.22 (95% CI; 0.09–0.38) of haploHCT, 0.21 (0.12–0.33) of MRD and 0.21 (0.13–0.30) of MUD-HCT. Accordingly, overall cause of death distribution at 2 years after transplant (Fig. 2a) shows a higher fraction of fatal infections after haploHCT (35.7%) compared to MRD (5.6%) and MUD transplantation (6.2%). The fraction of death due to relapse was lower after haploHCT (50%) than after MRD (88.9%) and MUD HCT (81.2%). Fatal, both acute or chronic GvHD and other reasons of death (including GF or SOS) were responsible for less than 10% of all deaths in all groups (GvHD; 7.1% of deaths after haploHCT vs. 5.6% after MRD-HCT and 6.2% after MUD-HCT, other reasons; 7.1% of deaths after haploHCT vs. 0% after MRD-HCT and 6.2% after MUD-HCT). Of Note, fatal SOS occurred only in 2 patients overall, both after MUD-HCT.

In the HaploHCT group, 4 out of 5 infectious deaths (*n* = 4/5) were caused by bacterial infections and 1 by uncontrolled fungal disease (*n* = 1/5). 3/5 deaths occurred before and 2/5 after neutrophil engraftment. In the MUD group (*n* = 2/2), both fatal infections were caused by bacterial sepsis after engraftment whereas after MRD transplantation (*n* = 1/1), the only fatal infection was due to a viral infection after neutrophil engraftment (cmv pneumonitis).

### Engraftment, chimerism and GvHD

**Fig. 3:**
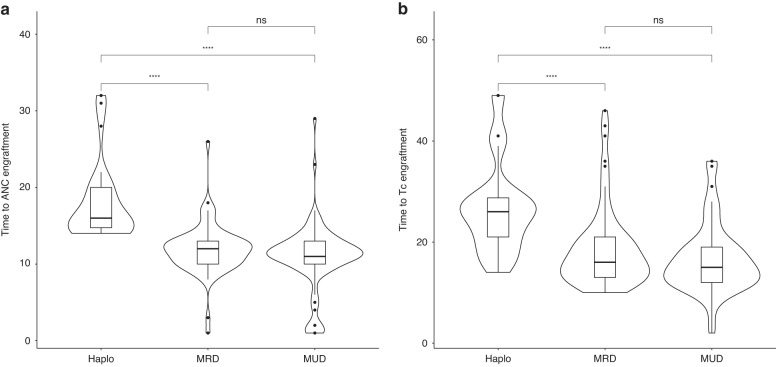
Days to engraftment after transplantation. **a** Neutrophil granulocytes. **b** Platelets (Tc). Violin plots highlight the distribution of values, boxes represents the 95% CI, horizontal lines represent means, and individual points show outliers. ****p < 0.05, ns non-significant.

Time to engraftment of neutrophils was significantly longer after haploHCT with a median of 16.0 days (interquartile range (IQR); 14.8–20.0) versus 12.0 days after MRD (IQR; 10.0–13.0) and 11.0 days after MUD-HCT (IQR; 10.0–13.0) as demonstrate in Table 2 and Fig. 3 (*p* < 0.001). Accordingly, median time to engraftment of platelets after haploHCT was 26.5 days (IQR; 21.2–36.8) versus 16.0 days (13.0–21.0) after MRD and 15.0 days (12.0–19.0) after MUD-HCT (*p* < 0.001).

**Table 2:** Outcome.

Outcomes	Donor Group			
	Haploidentical, *N* = 40^a^	MRD, *N* = 68^a^	MUD, *N* = 105^a^	*p*-value^b^
Follow-Up (m)	14.5 (6.5, 29.5)	19.6 (9.3, 40.6)	21.3 (11.4, 45.2)	
Death, overall	14 (35.0%)	20 (29.4%)	32 (30.5%)	ns
Relapse/Progression, overall	11 (27.5%)	28 (41.2%)	33 (31.4%)	ns
Time to Progression (m)	5.8 (3.1, 10.6)	6.0 (3.2, 9.4)	3.4 (3.0, 7.0)	ns
Outcome at Day 100				
Time to neutrophil-Engraftment >1 × 10^9/L (d)	16.0 (14.8, 20.0)	12.0 (10.0, 13.0)	11.0 (10.0, 13.0)	<0.001
Time to platelet engraftment >50 × 10^9/L (d)	26.5 (21.2, 36.8)	16.0 (13.0, 21.0)	15.0 (12.0, 19.0)	<0.001
Chimerism BM (%)	100.0 (100.0, 100.0)	100.0 (99.0, 100.0)	100.0 (99.0, 100.0)	ns
missing	10 (25%)	13 (19.1%)	25 (23.8%)	
aGvHD Grade ≥2	11 (27.5%)	10 (14.7%)	23 (21.9%)	ns
missing	1 (2.5%)	1 (1.5%)	2 (1.9%)	

Outcome at Day 730	Cumulative Incidence, % (95% CI)			
Overall Survival				
Cum. Inc. (95% CI)	0.59 (0.44, 0.79)	0.77 (0.67, 0.88)	0.72 (0.64, 0.82)	Log-rank; ns
HR (95% CI)	-	0.62 (0.32–1.19)	0.54 (0.26–1.14)	
Progression Free Survival				
Cum. Inc. (95% CI)	0.60 (0.46, 0.78)	0.55 (0.44, 0.69)	0.64 (0.55, 0.74)	Log-rank; ns
HR (95% CI)	-	0.87 (0.48–1.59)	1.08 (0.58–2.03)	
Non-Relapse Mortality				
Cum. Inc. (95% CI)	0.18 (0.08, 0.33)	0.029 (0.005, 0.09)	0.06 (0.02–0.12)	Gray’s test; 0.01
HR (95% CI)	-	0.31 (0.10–0.92)	0.31 (0.03–0.80)	

*ANC* absolute neutrophil count, *Tc* thrombocytes, *BM* bone marrow, *GvHD* graft versus host disease, *IQR* inter quartile range, *ns* non-significant, *HR* hazard ratio for an event vs. haploHCT, *CI* confidence interval, *Cum. Inc.* cumulative incidence.

^a^Median (IQR); *n* (%).

^b^Kruskal-Wallis rank sum test; Pearson’s Chi-squared test; Fisher’s exact test.

Follow-up Table 2 shows outcomes at day +100 and +730 (2 years) of follow-up. Until day +100, all cohorts reached a median 100% peripheral whole blood donor chimerism. At that time, cumulative incidence of acute GvHD grade II or higher was not statistically different after MRD-HCT (14.7%) compared to MUD-HCT (21.9%) and haploidentical HCT (27.5%, *p* = 0.2).

## Discussion

The results of our single-center retrospective analysis including 213 patients with AML and MDS show no significant differences in OS and PFS but significantly higher NRM after haploHCT performed with ptCY compared to HCT from MRD and MUD performed with conventional immunosuppression. We demonstrate higher NRM is driven predominantly by a higher cumulative incidence of fatal infections, with most fatal infections occurring early after transplantation. Meanwhile, rates of death due to progression or other transplant-related causes, such as fatal GvHD, were comparably low in all groups. Our findings are important because they provide real-life confirmation for AML/MDS of what has been shown in reference studies [10, 12, 14] and therefore support the efficacy of this widely adopted regimen for haploidentical HCT. Our results appear to be representative since median OS and cumulative incidence of causes of death among groups are similar to those recently published by Mehta and colleagues [14]. However, unlike us, they demonstrate significant differences in OS between haploHCT and matched donor transplantation in an overall larger cohort.

Although other analyses have not come to the same conclusions [10, 12, 23], higher rates of NRM and fatal infections after haploHCT have been demonstrated in several studies [13, 14] and its reasons and mechanisms are a widely discussed topic.

One important factor may be impaired infection control due to slower donor-derived CD4+ T-cell reconstitution after haploHCT. Compared to ptCY-based transplantation from MRD, McCurdy et al. demonstrated slower recovery of CD4 + T-cells early post-transplant but not of CD8+, B- or NK-cells after haploHCT [24]. Furthermore, in our population, ptCY was administred only after haploHCT resulting in further T-cell suppression [14, 25–27].

Second, as supported by our analysis, another major reason may be the longer time to neutrophil engraftment. Although only incompletely understood, delayed engraftment is thought to lead to an increased risk of early bacterial and fungal infections [14, 23, 28]. Notably, delayed engraftment was observed in our analysis even though the proportion of PBSC grafts was high in all groups, suggesting that graft source is not the major cause for slower engraftment, as hypothesized by others [10, 23, 29]. Furthermore, although MUDs were slightly younger than haploidentical donors, we also cannot clearly associate delayed engraftment with higher donor age, since similar age differences between MRD and MUD did not result in the same trend. This is relevant because several studies have suggested an adverse outcome after transplantation from older unrelated donors [30–33].

Third, as a higher rate of GvHD has been historically attributed to HLA-mismatched transplantations [2, 3, 34], the reluctance to decrease systemic immunosuppression post-transplant may have led to delayed weaning in our cohort, thereby promoting infectious complications. Among possible implications of our findings, we hypothesize that a less intensive immunosuppression protocol after haploHCT could lead to an even better outcome through lower NRM. Although previously suggested, until today this concept has only been tested rigorously in matched donor transplantation [35–38]. Since the rate of fatal GvHD was low in all groups of our analysis, the study of earlier IS cessation could be justified also after haploHCT.

Furthermore, antimicrobial prophylaxis regimens could be modified to more effectively prevent infections, most importantly early post-transplant. However, as no universally applicable infection control protocols have been published to date and regimens vary considerably between centers worldwide, there are no universally accepted recommendations due to lack of comparability [15]. These efforts are further complicated by the different microbial resistance profiles in different parts of the world.

We conclude that haploHCT is safe and effective in the treatment of AML and MDS with similar rates of OS and PFS. The higher NRM is caused mainly by a higher incidence of fatal infections which could be favored by the longer time to neutrophil engraftment after haploHCT.

The strengths of our analysis, although not as comprehensive as many registry-based analyses, lie in the completeness of the data and the homogeneity of the transplant protocols and patient groups with its focus on AML/MDS, as well as the long observation period. We demonstrate a very similar distribution of disease risk between the groups according to the only recently published ELN2022 risk classification [39]. In contrast to reference studies [10, 12, 14, 23], the majority of patients we compared underwent transplantation with PBSC grafts, which allowed us to minimise a potential confounder of outcome measures.

## Limitations

Apart from the above and the fact that this is a single-center, retrospective analysis, this study has several other limitations. Importantly, non-lethal infections were not systematically recorded. Within groups, different conditioning regimens were used and baseline characteristics of included patients vary slightly between groups. Furthermore, no systematic GvHD assessment after day +100 was documented, making it impossible to report GvHD-free survival as others have. In addition, fewer patients with MDS were included in the haploHCT group possibly altering its outcome, presumably because more time was available to find a MUD in these patients.

## Data Availability

All data can be made available upon reasonable request to the corresponding author.
